# Supplementary material for: Challenges Affecting Access to Health and Social Care Resources and Time Management among Parents of Children with Rett Syndrome: A Qualitative Case Study
Source: Int J Environ Res Public Health. 2020 Jun 21;17(12):4466. doi: 10.3390/ijerph17124466 (PMC7345745; doi:10.3390/ijerph17124466)
Supplement: Supplementary file 1 [file ijerph-17-04466-s001.pdf]

Supplemental Material Table S1. Example of Inductive codification process. Theme: Bureaucracy and social care.

Participant´s Narratives	Meaningful units	Meaningful groups	Theme
"It is difficult to avoid bureaucracy, it is mandatory to face it... It is a continuous confrontation, there is no rest... You cannot avoid feeling mistreated or misunderstood, we are forgotten as soon as we leave, we and the needs of our daughters... Bureaucracy is manifested in the position of the official who attends to you, and in my case, it always ends up in arguments..."<br><br>*"The bureaucratic procedures are exhausting. You have to submit more and more documents and when you think all the papers have been submitted, there is always one missing. Bureaucracy does not take into account our needs, and waiting times are getting longer"*	Dealing with a lot of bureaucracy<br>Bureaucracy is "a continuous struggle"<br>Feeling forgotten and mistreated<br>Confronted by the official administration employees	Fighting bureaucracy	Bureaucracy and social care
"Of course we felt the crisis. In the following years we saw many grants reduced or cancelled from public funds. Do you know who took advantage of this? The private centers, covering that gap with the famous early intervention centers. Many families can't afford to pay for the cost of their therapies."<br><br>*"The cost of health care is very high and we hardly have any help for our children's therapies anymore... It is normal that private centers charge for their services, but there should be more help to be able to afford it".*	A change after the economic crisis<br>Public aids have been reduced and private entities replace the public services<br>Unable to afford private services<br>Early intervention centers charge for their services<br>Parents cannot access early intervention<br>Home care is very expensive	Privatization of social care	

Supplemental Material Table S2. Demographic and clinical features of the family participants.

Family Code	Participant Code	Age (years)	Gender of participant	Number of children with RS	Age (years) of child with RS	Gender of child with RS	Age (months) when first symptoms were identified	Age (years/months) at diagnosis
FAM1	P1	63	Female	1	4	Female	12	2y
	P2	33	Female					
	P3	35	Male					
FAM2	P4	45	Female	1	9	Female	15	2y
	P5	48	Male					
FAM3	P6	38	Female	1	6	Female	12	3y 10m
	P7	43	Male					
FAM4	P8	45	Female	1	11	Male	3	3y

	P9	46	Male					
FAM5	P10	42	Female	1	5	Male	9	2y 6m
FAM6	P11	46	Female	1	11	Male	At birth (microcephaly)	10y
	P12	49	Male					
FAM7	P13	37	Female	1	12	Male	4	7y
	P14	42	Male					
FAM8	P15	46	Female	1	14	Male	At birth (microcephaly)	8y
	P16	48	Male					
FAM9	P17	41	Male	1	5	Female	10	1y 8m
	P18	36	Female					
FAM10	P19	39	Male	1	12	Female	Prenatal detection	3y
	P20	36	Female					
FAM11	P21	55	Female	1	16	Female	14	4y
FAM12	P22	63	Female	1	31	Female	At birth	4y

	P23	67	Male					
FAM13	P24	43	Male	1	17	Female	10	2y 6m
FAM14	P25	67	Male	1	38	Female	8	16y clinical dx<br>22y genetic dx
	P26	65	Female					
FAM15	P27	53	Male	1	19	Female	12	1y 6m
FAM16	P28	46	Male	1	12	Female	12	1y 6m
FAM17	P29	35	Female	1	7	Female	12	1y 6m
FAM18	P30	30	Female	1	7	Female	15	1y 6m
FAM19	P31	25	Female	1	3	Female	12	1y 8m

y= years; m= months; dx: diagnosis.
